# Comparison of Electrocardiogram Characteristics of Two Commercially Available Implantable Loop Monitors: Impact of These Characteristics in the Correct Adjudication of Recorded Events and Minimized Undersensing and Oversensing of Events

**DOI:** 10.19102/icrm.2025.16013

**Published:** 2025-01-15

**Authors:** Atul Prakash, Eisha Gupta, Tariq Hadaya, Ravnit Singh

**Affiliations:** 1 Department of Medicine and Cardiology, St Mary’s General Hospital, Passaic, NJ, USA

**Keywords:** ECG recordings, event monitoring, implantable loop monitors

## Abstract

Implantable cardiac monitors (ICMs) are useful in the detection of tachycardias, bradycardias, and atrial fibrillation. Undersensing and oversensing of events occur despite complex algorithms. The devices available have subtle differences, which may account for a difference in recorded characteristics. The electrocardiogram (ECG) characteristics of different monitors may influence their ability to correctly identify the events recorded. The objective is to compare the ECG characteristics of two commercially available implantable loop monitors and the ability to improve diagnostic accuracy. Two cohorts of patients were examined. Cohort 1 consisted of 30 patients with a Reveal LINQ I (Medtronic, Minneapolis, MN, USA) implanted, which was replaced with a BIOMONITOR III (Biotronik, Berlin, Germany) when the former had reached end of life. The new monitor was implanted at the same site in all patients. This provided a unique opportunity to compare ECGs obtained by both devices with no other confounding variable. Cohort 2 consisted of patients who had undergone implantation of either device at the discretion of the physician. This was therefore a retrospective analysis of 106 patients who had been implanted with one of the devices within a 2-year period. In both cohorts, we compared R-wave amplitude, the ability to accurately detect P-waves, and the frequency of undersensing and oversensing of events. In cohort 1, the mean R-wave was 0.35 ± 0.2 mV with the LINQ I as compared to 0.98 ± 0.4 with the BIOMONITOR III (*P* = .001). A P-wave in sinus rhythm was present in 19 (63%) subjects with the LINQ I implants versus 28 (93%) with the BIOMONITOR III implants (*P* = .004). Undersensing of events was noted in five (16%) patients with the LINQ I versus five (16%) with the BIOMONITOR III (*P* > .5). Oversensing was seen in 4 patients (13.33%) with the LINQ I versus 0 with the BIOMONITOR III (*P* = .012). In cohort 2, the mean R-wave with the BIOMONITOR III was significantly greater than with the LINQ I (0.65 ± 0.37 vs. 0.48 ± 0.38; *P* = .02). A visible P-wave was seen in 33 of 53 patients with the BIOMONITOR III as compared to 16 of 536 patients with the LINQ I monitor (*P* = .01). The number of patients identified as having under- or oversensing was, however, not significantly different (*P* = .08) in this cohort. In both patient cohorts, the BIOMONITOR III was noted to have significantly greater R-wave amplitude as compared with the LINQ I. A visible P-wave was also more commonly seen in patients with a BIOMONITOR III. In the paired cohort, but not in the unpaired cohort, the BIOMONITOR III was less likely to oversense R-waves. There was no significant difference in undersensing between the devices.

## Introduction

Event monitoring has evolved significantly over the last three decades.

From external wearable monitors to implantable devices, this technology has aided in the detection of rhythm abnormalities along with the estimation of their burden. Implantable cardiac monitors (ICMs) have specifically been used when the frequency of events is low; the events have an abrupt onset; and there is a need for robust, reliable monitoring without gaps.^1^

ICMs have simultaneously decreased in size while boasting increased memory and battery life.^2^ Despite this, undersensing and oversensing remain challenges. Several algorithms have been developed to minimize these phenomena.^3^ Irregularity of R–R intervals is used for the diagnosis of atrial fibrillation (AF).^4,5^ Tachycardic events are still difficult to classify with just regularity and morphology. The distinction between supraventricular tachycardia, ventricular tachycardia, and atrial flutter with 1:1 or 2:1 conduction is still difficult without a discernible P-wave. Despite the auto-gain function in ICMs, dropouts are still being classified as asystole by these devices.^6^ For implantable pacemakers and defibrillators, the presence of an atrial electrogram (EGM) is still very valuable for the final adjudication of a tachycardia event.^7^

### Hypothesis

For this study, we hypothesized that the amplitude of the recorded R-wave would reduce R-wave undersensing and therefore false-positive bradycardia events, while the presence of a visible P-wave would help adjudicate the true tachyarrhythmia events.

### Aims and objectives

We sought to (1) compare the ECG characteristics obtained in two commercially available ICMs and (2) evaluate whether this difference could reduce the undersensing and oversensing and help correctly characterize the tachycardia and bradycardia episodes.

## Methods

### Study design

In this study, we compared the ECG characteristics of two implantable monitors—the Reveal LINQ I (Medtronic, Minneapolis, MN, USA) and the BIOMONITOR III (Biotronik, Berlin, Germany).

The study analysis was conducted using two cohorts of patients.

#### Cohort 1

Patients implanted with a Reveal LINQ I monitor (Medtronic) subsequently received a BIOMONITOR III (Biotronik) when the initial ICM had reached the elective replacement indicator. The BIOMONITOR III was implanted at the same site as the explanted LINQ I monitor. A new loop was implanted as it was felt that the clinical indication for monitoring was still present. The same site allowed the elimination of confounding patient variables in the comparison of the two monitors. The LINQ II device (Medtronic) was not available at our institution.

#### Cohort 2

Patients were implanted with either a Reveal LINQ I monitor or a BIOMONITOR III over the span of a 2-year period (2021–2022). A comparison was made retrospectively in these two groups of patients.

### Device characteristics

The Reveal LINQ I is a leadless loop recorder sized at 44.8 mm × 7.2 mm × 4.0 mm, with a specific mass of 2.5 ± 0.5 g and a 37.7-mm distance between the electrodes.^8^

The BIOMONITOR III is a leadless implantable loop recorder (ILR) that is sized at 77.5 mm × 8.3 mm × 4.3 mm, with a specific mass of 5.0 ± 0.5 g, and the recording dipole is 77.5 mm, including an antenna.^9^

The devices were inserted subcutaneously under local anesthesia in the same location in the cohort 1 patients and standard locations in the cohort 2 patients.

The two devices offer remote monitoring, and the arrhythmia detection is based on algorithms that use the identification of the QRS signals.

The detection of atrial fibrillation is based on an algorithm that analyzes the R–R interval variability/stability, which depends on the QRS cycle differences. The algorithm detects the onset of an AF episode when the programmable threshold is exceeded.

### Data analysis

The ECG recordings were studied, and the following parameters were analyzed: (1) amplitude of the R-wave (noted at the time of implant), (2) presence of a visible P-wave in sinus rhythm, and (3) episodes of undersensing and oversensing.

The presence or absence of a P-wave and adjudication of under- and oversensing were initially reported by the patients’ electrophysiologist and then validated by a electrophysiologist who was blinded regarding the monitor type or any other information. The second electrophysiologist was not a part of the study/analysis.

### Determination of a visible P-wave

The ECG recordings were analyzed manually by an independent investigator blinded to loop recorder type and patient information, and the presence or absence of a P-wave was recorded for each patient.

### Determination of oversensing and undersensing episodes

The ECG recordings were analyzed manually for R–R variability, and the determination of oversensing and undersensing episodes was recorded.

### Statistical analysis

All data analysis and hypothesis testing were performed using R Statistical Software (R Foundation for Statistical Computing, Vienna, Austria).

A paired *t* test was used to look for a mean difference in R-wave amplitude between the two sets of observations for cohort 1; for cohort 2, a standard *t* test was used.

A chi-squared statistical test was used in both cohorts to test for an association between the presence of P-waves and the type of monitor.

## RESULTS

### Study population

#### Cohort 1

A total of 30 patients who initially had Reveal LINQ I monitor subsequently received the BIOMONITOR III as a replacement.

**Figure 1:**
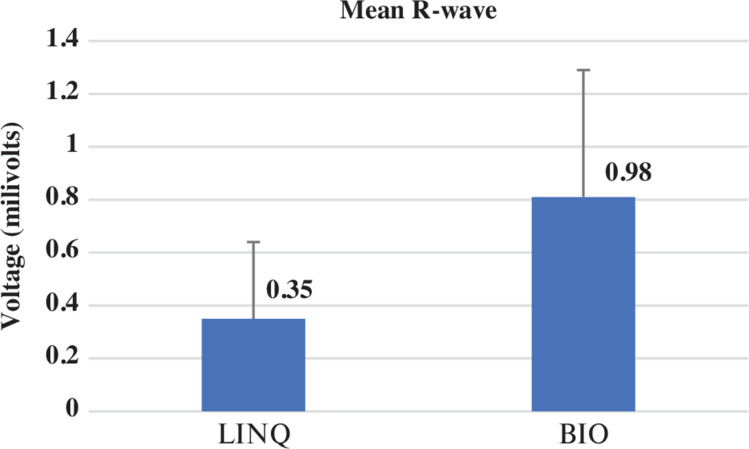
Comparison of mean R-waves between the Reveal LINQ I and BIOMONITOR III (*P* = .001). Bar diagram showing the mean R-wave with the LINQ I (0.35 ± 0.2 mV) or BIOMONITOR III (0.98 ± 0.4 mV).

The mean age of these patients was 70 ± 7.34 years. There were 17 men and 13 women in the studied population, and their average body mass index (BMI) was 28.4 ± 5.05 kg/m^2^. Twelve-lead ECGs for these patients had the tallest R-wave in the frontal plane leads ranging from 0.4–1.6 mV, with a mean of 0.62 ± 0.27 mV. Left ventricular hypertrophy as per voltage criteria was noted in 5 of the 30 patients, while left bundle branch block was observed in 4 patients and right bundle branch block was seen in 1 patient. The indication of the implant was the management of AF in 18 and cryptogenic stroke in 5 patients, while, in 7 patients, the indication was syncope. The mean follow-up with the LINQ I monitor was 38 ± 6 months, whereas the follow-up with the BIOMONITOR III was 26 ± 7 months. The mean R-wave was 0.35 ± 0.2 mV with the LINQ I as compared to 0.98 ± 0.4 mV with the BIOMONITOR III (*P* = .001) **(Figure 1)**.

**Figure 2:**
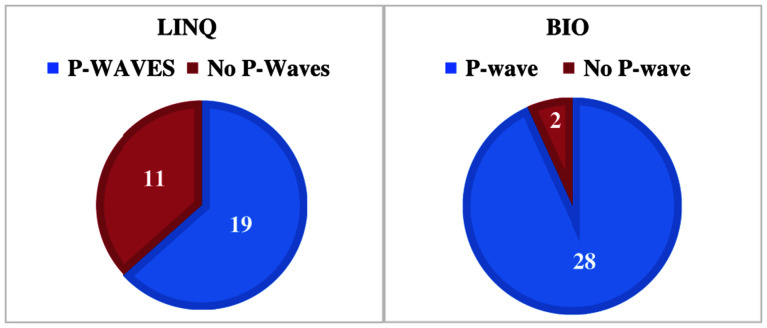
Comparison of the presence of P-waves between the Reveal LINQ I and BIOMONITOR III in cohort 1 (*P* = .004). Pie chart showing the presence of visible P-waves with the LINQ I (19/30 patients) and BIOMONITOR III (28/30 patients).

The P-wave in sinus rhythm was present in 19 (63%) subjects with the LINQ I implant versus 28 (93%) patients with the BIOMONITOR III implant (*P* = .004) **(Figure 2)**.

Undersensing of events was noted in 5 (16%) patients with the LINQ I versus 5 (16%) with the BIOMONITOR III. Undersensing was classified as falsely reported bradycardia or asystole by the ILR. The undersensing resulted from the T-wave, resulting in the undersensing of the subsequent R-waves as well as the r-waves. Oversensing was seen in 4 (13.33%) patients with the LINQ I versus 0 with the BIOMONITOR III (*P* = .012). The oversensing was because of intermittent noise in all four cases. Despite reprogramming based on the available algorithms, both undersensing and oversensing could not be resolved in any patients. Twelve of the 30 patients had true AF, which was confirmed by the absence of the P-waves. The presence of a P-wave was decisive in the correct adjudication of the AF episodes.

The amplitude of the R-wave and a visible P-wave had a negative correlation with BMI (*r* = −0.2). The presence of the P-wave helped in the correct diagnosis of both bradycardic events and tachycardic events.

#### Cohort 2

These patients were divided into two groups according to their implants: group 1 patients had a Reveal LINQ I, while group 2 patients had a BIOMONITOR III. A total of 106 patients were included in this analysis, with 53 (50%) patients having a LINQ I (group 1) and 53 (50%) having a BIOMONITOR III (group 2). The baseline characteristics were well balanced between the two groups.

In group 1, there were 19 men and 34 women. The mean BMI was 28.2 ± 5.35 kg/m^2^. The indication for implantation was possible AF in 38 and syncope in 15. The mean age was 74.6 ± 10.75 years. The mean R-wave was 0.48 ± 0.38 mV. Visible P-waves were noted in 16 (30.18%) patients. Oversensing was noted in 8 (15.09%) patients, while undersensing was noted in 3 (5.66%) patients **(Table 1)**.

**Table 1:** Comparison of Demographic Data Between Study Groups

	Group 1 (Reveal LINQ I)	Group 2 (BIOMONITOR III)
*N*	53	53
Mean age (years)	74.6 ± 10.75	72.94 ± 10.42
Sex		
Female	34	26
Male	19	27
Indication		
AF	38	37
Syncope	15	16
BMI kg/m^2^	28.2 ± 5.35	29.20 ± 5.35

*Abbreviations:* AF, atrial fibrillation; BMI, body mass index. Values are expressed as *N* (number).

In group 2, there were 27 men and 26 women. The mean BMI was 29.20 ± 5.35 kg/m^2^. The indication for implantation was AF in 37 and syncope in 16. The mean age was 72.94 ± 10.42 years. The mean R-wave was 0.65 ± 0.37 mV. Visible P-waves were noted in 33 (62.26%) patients. Oversensing was noted in 8 (15.09%) patients and undersensing was noted in 0 (0%) patients **(Table 1)**.

**Figure 3:**
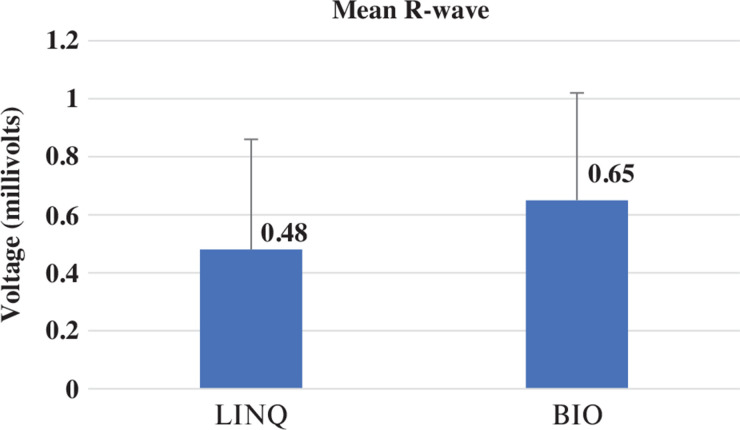
Comparison of the mean R-wave in group 1 (Reveal LINQ I) and group 2 (BIOMONITOR III) (*P* = .02). Bar diagram showing the mean R-wave in group 1 (0.48 ± 0.3 mV) and in group 2 (0.65 ± 0.3 mV).

**Figure 4:**
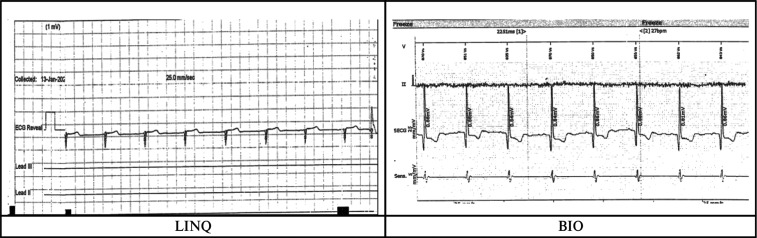
Electrograms from the Reveal LINQ I and BIOMONITOR III monitors in the same patient after replacement. The electrogram of the device at the same location in the same patient with a greater amplitude R-wave and a visible P-wave seen with the BIOMONITOR III is not well visualized with the LINQ I.

**Figure 5:**
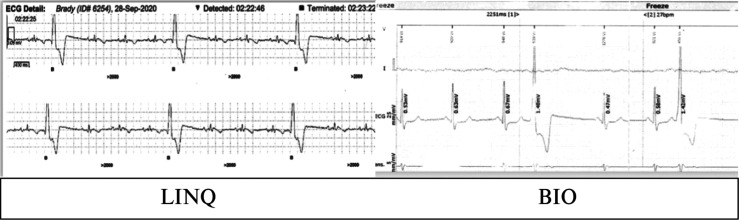
Electrogram comparison between the Reveal LINQ I and BIOMONITOR III for undersensing. Following the premature ventricular complex, undersensing is noted in the LINQ I electrogram, whereas no undersensing is noted after the premature ventricular complex with the BIOMONITOR III.

The measured R-wave was significantly greater in group 2 as compared to group 1 **(Figure 3)**. The presence of a P-wave in sinus rhythm was compared between groups; a greater number of individuals had a visible P-wave in group 2 as compared to group 1 (*P* = .009) **(Figure 4)**. Undersensing and oversensing for events, however, were not significantly different between the two groups (*P* = .08). **Figure 4** presents an example of an ECG in a patient with the Reveal LINQ I and BIOMONITOR III. **Figure 5** shows undersensing after a premature ventricular complex with a LINQ I monitor but not with the BIOMONITOR III.

## Discussion

ICMs continue to evolve with complex algorithms aimed at increasing sensitivity and specificity in detecting rhythm disturbances. Despite this, undersensing and oversensing issues persist.^6,10^ The relationship of the P-wave and the R-wave is at times critical to the final diagnosis of a recorded event.^11^ Bradycardia events as recorded by the monitor are at times a result of undersensing of the R-wave.^12–14^ Even dynamic patient factors such as body posture can affect the R-wave amplitude.^15^

In our study, we found that many undersensing events occurred due to premature ventricular complexes and large T-waves resulting in failure to sense the following R-wave. Oversensing was a result of noise. The incorrect classification of a tachycardic event that was correctly adjudicated when the event was analyzed with a P-wave (if present) was not labeled as oversensing.

In our first cohort, there was a unique opportunity to have patients act as their own controls, allowing for a robust comparison of the R-waves between two commercially available loop monitors: the Reveal LINQ I and the BIOMONITOR III. The greater R-wave seen with the BIOMONITOR III was likely due to the larger surface area of its recording dipole (77 vs. 37.7 mm); the presence of a discernible P-wave may well have resulted from this as well. As might be expected, the size of the R-wave had a negative correlation with BMI. Though the incidence of oversensing was less with the larger device, the greater R-wave surprisingly did not reduce the undersensing events in this admittedly small sample size.

All loop monitors continue to develop these recording capabilities with increasing sensing technology. Undoubtedly, the complex detection algorithms do help reduce false-positive events.^16^ Despite these innovative algorithms, we still require a visible P-wave and a reasonable amplitude of the ventricular recording. This is very similar to the final adjudication of a tachycardia event with implantable pacemakers or defibrillators, where the availability of both atrial and ventricular recordings is very helpful.

The recommended locations for these loop monitors are on the basis of non-individualized data. We would emphasize that, based on this study, algorithms to increase the sensitivity and specificity do not compensate for the presence of a visible P-wave and the amplitude of the R-wave. The site of monitor implantation should be based on careful mapping as multiple factors can affect the quality of the recordings, especially in those with a higher BMI.

### Limitations

The biggest limitation of our study is its smaller sample size, leading to limited power in comparison to instances of undersensing/oversensing. In addition, in the first cohort, loop monitor placement always followed the same sequence of the LINQ I being replaced by the BIOMONITOR III, making it unclear if there was also a temporal effect impacting the quality of recordings. Also, the sample size of the first cohort may account for a lack of correlation of the R-wave amplitude and the under- and oversensing of events. The visible P-wave and the amplitude did help in the adjudication of the events recorded. The second cohort is a retrospective analysis that does not involve true randomization and is, of course, subject to confounding by unknown factors. The third limitation is the unequal follow-up of patients in cohort 1, which may influence the magnitude of the number of under- and oversensed events though not the patients with the same and not the characteristics of the R-wave and P-wave. The unequal follow-up results from the fact that the BIOMONITOR III has not reached end of life yet.

## References

[1] Bisignani A, De Bonis S, Mancuso L, Ceravolo G, Bisignani G (2018). Implantable loop recorder in clinical practice. J Arrhythm.

[2] Task Force members; Brignoles M, Vardas P, Hoffman E (2009). Indications for the use of diagnostic implantable and external ECG loop recorders. Europace.

[3] Dwivedi A, Joza J, Malkani K (2018). Implantable loop recorder in inherited arrhythmia diseases: a critical tool for symptom diagnosis and advanced risk stratification. JACC Clin Electrophysiol.

[4] Sandesara CM, Gopinathannair R, Olshansky B (2017). Implantable cardiac monitors: evolution through disruption. J Innov Cardiac Rhythm Manag.

[5] Lian J, Wang L, Muessig D (2011). A simple method to detect atrial fibrillation using RR intervals. Am J Cardiol.

[6] Chrysostomakis SI, Klapsinos NC, Simantirakis EN, Marketou ME, Kambouraki DC, Vardas PE (2003). Sensing issues related to the clinical use of implantable loop recorders. Europace.

[7] Podd SJ, Sugihara C, Furniss SS, Sulke N (2016). Are implantable cardiac monitors the ‘gold standard’ for atrial fibrillation detection? A prospective randomized trial comparing atrial fibrillation monitoring using implantable cardiac monitors and DDDRP permanent pacemakers in post atrial fibrillation ablation patients. Europace.

[8] Medtronic Reveal LINQ Product Specifications. Insertable cardiac monitoring system.

[9] Biotronik Technical Manual Biomonitor III.

[10] Rav Acha M, Soifer E, Hasin T (2020). Cardiac implantable electronic miniaturized and micro devices. Micromachines (Basel).

[11] Ciconte G, Giacopelli D, Pappone C (2017). The role of implantable cardiac monitors in atrial fibrillation management. J Atr Fibrillation.

[12] De Coster M, Demolder A, De Meyer V, Vandenbulcke F, Van Heuverswyn F, De Pooter J (2020). Diagnostic accuracy of R-wave detection by insertable cardiac monitors. Pacing Clin Electrophysiol.

[13] Guarracini F, Testolina M, Giacopelli D (2022). Programming optimization in implantable cardiac monitors to reduce false-positive arrhythmia alerts: a call for research. Diagnostics (Basel).

[14] Russo V, Covino S, De Pasquale V (2023). Remote monitoring of implantable cardiac monitors in patients with unexplained syncope: predictors of false-positive alert episodes. Pacing Clin Electrophysiol.

[15] Swale M, Paul V, Delacroix S (2022). Changes in R-wave amplitude at implantation are associated with gender and orientation of insertable cardiac monitor: observations from the confirm Rx™ body posture and physical activity study. BMC Cardiovasc Disord.

[16] Maines M, Degiampietro M, Tomasi G (2023). Strategic reprogramming of implantable cardiac monitors reduces the false-positive remote alert burden in a nurse-led service. Eur J Cardiovasc Nurs.

